# Agriculture Development, Pesticide Application and Its Impact on the Environment

**DOI:** 10.3390/ijerph18031112

**Published:** 2021-01-27

**Authors:** Muyesaier Tudi, Huada Daniel Ruan, Li Wang, Jia Lyu, Ross Sadler, Des Connell, Cordia Chu, Dung Tri Phung

**Affiliations:** 1 Key Laboratory of Land Surface Pattern and Simulation, Institute of Geographical Sciences and Natural Resources Research, Chinese Academy of Sciences, No. 11 Datun Road, Beijing 100101, China; muyesaier.tudi@griffithuni.edu.au (M.T.); lvjia@nieh.chinacdc.cn (J.L.); 2 Centre for Environment and Population Health, School of Medicine, Griffith University, 170 Kessels Road, Nathan, QLD 4111, Australia; hruan@uic.edu.cn (H.D.R.); ross.sadler@griffith.edu.au (R.S.); c.chu@griffith.edu.au (C.C.); d.phung@griffith.edu.au (D.T.P.); 3 Environmental Science Program, Beijing Normal University-Hong Kong Baptist University United International College, 2000 Jintong Road, Tangjiawan, Zhuhai 519080, China; 4 Faculty of Health, Medicine and Life Sciences, Maastricht University, 6200 MD Maastricht, The Netherlands; 5 China CDC Key Laboratory of Environment and Population Health, National Institute of Environmental Health, Chinese Center for Disease Control and Prevention, No. 29 Nanwei Road, Beijing 100050, China; 6 School of Environment and Science, Griffith University, 170 Kessel Road, Nathan, QLD 4111, Australia; d.connell@griffith.edu.au

**Keywords:** agriculture, environment, review pesticide

## Abstract

Pesticides are indispensable in agricultural production. They have been used by farmers to control weeds and insects, and their remarkable increases in agricultural products have been reported. The increase in the world’s population in the 20th century could not have been possible without a parallel increase in food production. About one-third of agricultural products are produced depending on the application of pesticides. Without the use of pesticides, there would be a 78% loss of fruit production, a 54% loss of vegetable production, and a 32% loss of cereal production. Therefore, pesticides play a critical role in reducing diseases and increasing crop yields worldwide. Thus, it is essential to discuss the agricultural development process; the historical perspective, types and specific uses of pesticides; and pesticide behavior, its contamination, and adverse effects on the natural environment. The review study indicates that agricultural development has a long history in many places around the world. The history of pesticide use can be divided into three periods of time. Pesticides are classified by different classification terms such as chemical classes, functional groups, modes of action, and toxicity. Pesticides are used to kill pests and control weeds using chemical ingredients; hence, they can also be toxic to other organisms, including birds, fish, beneficial insects, and non-target plants, as well as air, water, soil, and crops. Moreover, pesticide contamination moves away from the target plants, resulting in environmental pollution. Such chemical residues impact human health through environmental and food contamination. In addition, climate change-related factors also impact on pesticide application and result in increased pesticide usage and pesticide pollution. Therefore, this review will provide the scientific information necessary for pesticide application and management in the future.

## 1. Introduction

The group of substances known as pesticides pertains to substances used as insecticides, fungicides, herbicides, rodenticides, molluscicides, and nematicides [1]. It is generally accepted that pesticides play an important role in agricultural development because they can reduce the losses of agricultural products and improve the affordable yield and quality of food [2,3,4]. Because of the urgency to improve food production and control insect-borne diseases, the development of pesticides increased during World War II (1939-1945). Additionaly, from the 1940s onwards, the increased use of synthetic crop protection chemicals permitted a further increase in food production [1]. Moreover, worldwide pesticide production increased at a rate of about 11% per year, from 0.2 million tons in the 1950s to more than 5 million tons by 2000 [5]. Three billion kilograms of pesticides are used worldwide every year [6], while only 1% of total pesticides are effectively used to control insect pests on target plants [1]. The large amounts of remaining pesticides penetrate or reach non-target plants and environmental media. As a consequence, pesticide contamination has polluted the environment and caused negative impacts on human health [1,7].

This literature review firstly provides basic scientific information about the agricultural development process, the historical perspective of pesticide usage, general types of pesticide in use, and the role of pesticides in agriculture. Specific focus is then put on pesticide behavior in the environment, climate change-related factors in pesticide use and its adverse effects on the natural environment. Finally this study provides a new direction for the application and management of pesticides.

## 2. Agricultural Development Process

Agricultural development has a long history in many places around the world. Agricultural practice began about 10,000 years ago in the Fertile Crescent of Mesopotamia, corresponding roughly to most of today’s Iraq, Turkey, Syria and Jordan [8]. People who lived in these areas collected edible seeds through means such as fire-stick farming, and forest gardening. When the population became more settled and lived on farms, large amounts of wheat, barley, peas, lentils, chickpeas, bitter vetch, and flax were cultivated [9]. Rice and sorghum were farmed in the Sahel region of Africa about 7500 years ago [10]. Davies (1968) furthermore indicates that some local crops were also domesticated independently in West Africa as well as in New Guinea and Ethiopia about 7500 years ago. Rice and millet were domesticated in China [11]. America independently domesticated corn, squashes, potato, and sunflowers [12]. The farmed crops often suffer from pests, weeds, and diseases which could result in a considerable loss in crop yield. Without pesticide usage, the loss of fruits, vegetables, and cereals due to pests and diseases would be as much as 78%, 54%, and 32%, respectively [13]. Therefore, there is an urgent need for scholars and the public to look for ways to overcome the problems caused by pests and diseases.

## 3. Historical Perspectives of Pesticide Usage

The history of pesticide use can be divided into three periods of time. During the first period before the 1870s, pests were controlled by using various natural compounds. The first recorded use of insecticides was about 4500 years ago by Sumerians [8]. They used sulfur compounds to control insects and mites. About 3200 years ago, the Chinese used mercury and arsenical compounds to control body lice. There was no chemical industry, so all products used were derived directly from readily available animal, plant, or mineral sources. For example, volatile substances were often applied by “smoking”. The principle was to burn straw, chaff, hedge clippings, crabs, fish, dung, or other animal products, so that the smoke, preferably malodorous, could spread throughout the orchard, crop, or vineyard [8]. It was generally assumed that such smoke would eliminate blight or mildew. Smoke was also used against insects. People controlled weeds mainly by hand weeding, while various chemical methods were also reported [14]. Pyrethrum is obtained from the dried flowers of the chrysanthemum *Cineraria folium*, “pyrethrum daisies”, and has been used as an insecticide for over 2000 years.

During the second period, between 1870 and 1945, people began to use inorganic synthetic materials. At the end of the 1800s, people in Sweden used copper and sulfur compounds against fungal attack in fruit and potatoes [15]. Since then, people have been using many inorganic chemicals, including the Bordeaux mixture, based on copper sulfate and lime arsenic, as pesticides, and they are still being used to prevent numerous fungal diseases [1].

The third period started after 1945 [8], represented by the use of synthetic pesticides with the discovery of the effects of Dichlorodiphenyltrichloroethane (DDT), β-Hexachlorocyclohexane (BHC), aldrin, dieldrin, endrin, chlordane, parathion, captan, and 2,4-D [16]. The disadvantages of many of these products were at their high rates of application, lack of selectivity, and high toxicity. For example, DDT was widely used all over the world since it had low toxicity to mammals, and it reduced insect-borne diseases, such as malaria, yellow fever, and typhus [17,18]. The book “Silent Spring” indicated the negative impacts of pesticides on the environment and human health. The book aroused great attention among scholars and the public [1]. DDT was banned in 1972 in the US because of its harm to non-target plants and animals, as well as problems with its significant ability to accumulate in tissues and persist, causing long-term damage [19]. Between the 1970s and 1990s, new families of chemicals, such as triazolopyrimidine, triketone and isoxazole herbicides, strobilurin and azolone fungicides, chloronicotinyl, spinosyn, fiprole diacylhydrazine, and organophosphate insecticides, have been introduced to the market and most of the new chemicals can be used in grams rather than kilograms per hectare [1,18].

In modern agriculture, scholars are trying to develop genetically engineered crops designed to produce their own insecticides or exhibit resistance to broad-spectrum herbicide products or pests. This new pest management could reduce chemical use and its negative impacts on the environment [1].

## 4. Types of Pesticide in Use

**Figure 1:**
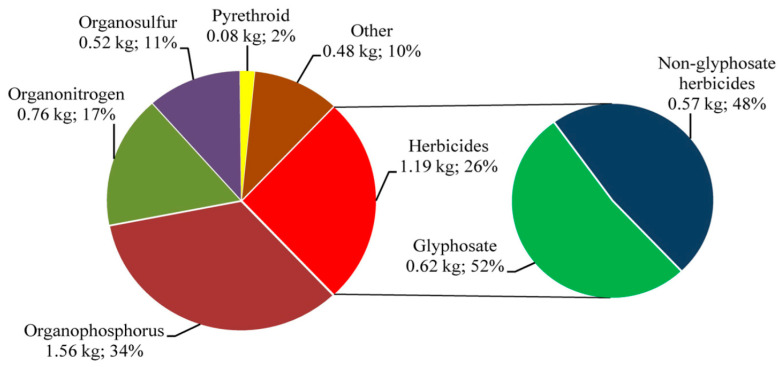
Summary of agricultural use of each class of pesticide in China [24].

Pesticides are classified by different classification terms such as chemical classes, functional groups, modes of action, and toxicity [20]. Firstly, pesticides are classified by different targets of pests, including fungicides, insecticides, herbicides, and rodenticides. For example, fungicides are used to kill fungi, insecticides are used to kill insects, while herbicides are used to kill weeds [21,22]. In terms of chemical classes, pesticides are classified into organic and inorganic ingredients. Inorganic pesticides include copper sulfate, ferrous sulfate, copper, lime, and sulfur. The ingredients of organic pesticides are more complicated [23]. Organic pesticides can be classified according to their chemical structure, such as chlorohydrocarbon insecticides, organophosphorus insecticides, carbamate insecticides, synthetic pyrethroid insecticides, metabolite and hormone analog herbicides, synthetic urea herbicides, triazine herbicides, benzimidazole nematocides, metaldehyde molluscicides, metal phosphide rodenticides, and D group vitamin-based rodenticides. Figure 1 shows the summary of the agricultural use of each class of pesticide in China [24].

## 5. The Role of Pesticides

Tremendous primary benefits have been achieved using different types of pesticides in a range of areas, including public health and agricultural activities [25]. In terms of public health, pesticides are used in daily life to kill pests, including mosquitoes, ticks, rats, and mice in houses, offices, malls, and streets. As a result, the immense burden of diseases caused by these vectors has been substantially reduced or eliminated [21,23,26]. Insecticides are often the most practical way to control insects that can spread deadly diseases such as malaria, possibly resulting in an estimated death count of 5000 deaths globally each day [17]. In addition, pesticides are indispensable in agricultural production. They have been used by farmers to control weeds and insects in agricultural cultivation, and remarkable increases in agricultural products have been reported as a result of pesticide use [1,27]. To cope with demographic growth, there has been a significant increase in agricultural yield since the beginning of the 20th century. Within one century, population growth increased from 1.5 billion in 1900 to about 6.1 billion in 2000, corresponding to a world population growth rate three times greater than during the entire history of humanity. Since 2003, the world’s population has increased by yet another billion, and given the current growth rates, it is projected to reach 9.4–10 billion by 2050 [5]. The increase in the world’s population in the 20th century could not have been possible without a parallel increase in food production. Although increases in food productivity have been due to several factors, including the use of chemicals, better plant varieties, and the use of machinery, pesticides have been an integral part of the process by reducing harvest losses caused by weeds, diseases, and insect pests [25]. About one-third of agricultural products are produced using pesticides. Without the use of pesticides, there would be a 78% loss of fruit production, a 54% loss of vegetable production, and a 32% loss of cereal production [27]. Therefore, pesticides play a critical role in reducing diseases and improving the increase in crop yields worldwide. Thus, they have made a significant contribution to alleviating hunger and providing access to an abundant supply of high-quality food.

There is also a secondary benefit from pesticide usage, which is less immediate and less intuitively obvious, with long-term consequences, including farm and agribusiness revenues, nutrition and health improvement, food safety, quality of life improvement, a wider range of viable crops, life expectancy increases, reduced vet and medical costs, a fitter population, stress, maintenance costs, soil erosion/moisture loss, greenhouse gas emission, international spread of diseases, global warming, increased export revenues, workforce productivity, biodiversity, and cropping due to agronomic consultation [28]. Controlling pests of pastures will bring significant livestock productivity benefits. For example, insecticide spraying may cost USD 10/ha to control red-legged earth mites in clover, however, sheep farmers in Australia can increase the value of their wool yield by USD 50/ha [29]. Another example is that increased agricultural productivity using proper pesticides may significantly increase farming families’ income [30]. The value of nutritious, safe, and affordable food contributes to life expectancy as a health promoter [28].

## 6. Pesticide Behavior in the Environment

**Figure 2:**
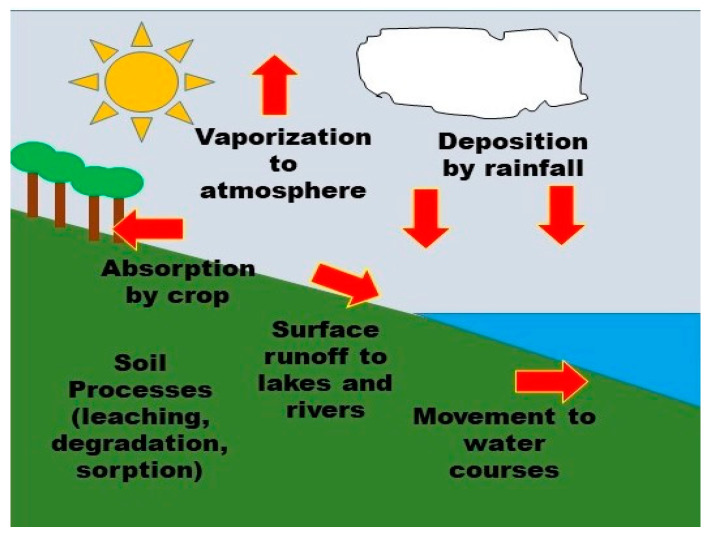
Pesticide behavior in the natural environment (by authors).

When pesticides are applied to a target plant or disposed of, they have the potential to enter the environment. On entering the environment, pesticides can undergo processes such as transfer (or movement) and degradation [31,32,33]. Pesticide degradation in the environment produces new chemicals [34]. Pesticides relocate from the target site to other environmental media or non-target plants by transfer processes including adsorption, leaching, volatilization, spray drift, and runoff (Figure 2) [35]. The different types of chemicals indicate their differences in environmental behavior. For example, organochlorine compounds such as DDT have low acute toxicity but show a significant ability to accumulate in tissues and persist in causing long-term damage. They have been banned from sale in most countries, but their residues remain in the environment for a long time because of their nature. While organophosphate pesticides are of low persistence, they have appreciable acute toxicity in mammals [23,36].

### 6.1. Pesticide Degradation

After pesticides are applied to the target organism, they are degraded by microbes, chemical reactions, or light [37]. Depending on the environmental conditions and the pesticide’s chemical characteristics [38], degradation may take from hours to days or even years [39]. Pesticide degradation processes control pesticide persistence in soils and yield different metabolites [40]. It also provides the concept of a half-life of the pesticides in the environment [34]. For example, in the case of chlorpyrifos, the major metabolite 3, 5, 6-trichloro-2-pyridinol (TCP) of chlorpyrifos is much more mobile and toxic than its parent chlorpyrifos [41]. Chlorpyrifos and its degradation products have been frequently detected in soils, sediments, and groundwater in many areas. These chemicals are considered to be endocrine-disrupting chemicals, possibly posing potential risks to human health [42].

There are three types of pesticide degradation [43,44]. Microbial degradation is the degradation of pesticides by microorganisms such as fungi and bacteria [45]. For example, biodegradation is the main path of niclosamide degradation in natural environments, as aerobatic and anaerobic naturalized microorganisms have a high capability to degrade niclosamide [43]. Factors including oxygen, temperature, soil moisture, soil pH, and soil porous structure influence pesticide microbial degradation [31,42,44,46]. For example, the enantioselective degradation of benalaxyl is mainly influenced by pH, with a greater degradation in soils with higher pH values [47].

Pesticides can be degraded by chemical reactions in the soil. This process is called chemical degradation [48]. Moreover, the chemical reaction of sunlight radiation plays an important role in the degradation of molecules on soil surfaces because it is always active [49]. The rate and type of chemical degradation are influenced by soil temperature, pH levels, moisture, and the binding of insecticides to the soil [31].

Photo-degradation is the degradation of pesticides by sunlight [50]. All insecticides are capable of photo-degradation to some extent, and the rate of degradation depends on the intensity of light, length of exposure, and the properties of the insecticide [31]. For example, niclosamide could hydrolyze to generate 5-chlorosalicylic acid and 2-chloro-4-nitroaniline under the effect of light [43].

### 6.2. Pesticide Migration

#### 6.2.1. Sorption

When pesticides are used, only a small amount of the applied pesticides displays a protective role to fight against plant diseases. In contrast, a large amount of pesticides reaches the soil, resulting in severe soil pollution [51,52]. The sorption process is a phenomenon that binds pesticides to soil particles due to the attraction between chemical and soil particles [51,53,54,55]. In addition, adsorption isotherms can be obtained according to the standard batch equilibration method (OECD106, 2000) and used for the assessment of pesticide retention in the environmental media [56].

Various factors influence this soil absorption process. Some soil factors, including pH, organic matter [42,53,57,58], and soil amendment [59], impact the adsorption of pesticides. Moreover, soils rich in organic matter or clay are much more adsorptive to pesticides than coarse, sandy soils, because clay or organic soils either have a greater particle surface area, or more sites onto which insecticides can be bound [45,55,60,61] For example, the adsorption and desorption abilities of endosulfan may be related to the contents of clay and organic matter in the soil [46,62]. The study shows the sorption/desorption and mobility of strobilurin fungicides in three Chinese soils in the order of Jiangxi red soil > Taihu paddy soil > Northeast China black soil. The main reasons for this result are soil properties, including organic matter (SOM), pH, and cationic exchange capacity (CEC) influencing the adsorption/desorption of the fungicides. Moisture also influences the adsorption of pesticides in the soil [31]. Generally, dry soils absorb more insecticides than wet soils because water molecules compete with the insecticides for the binding sites in wet soils. Temperature is another factor that influences ammonium nitrogen adsorption [63]. The humic acid colloid also influences the adsorption of DDT in sediments [59,64].

Since some pesticides have a long persistency in the soil [59,65], they can be absorbed by plants during their growth. Such types of pesticides could damage or leave residues in crops [66,67,68,69], Positively charged pesticide molecules are attracted to negatively charged clay particles and can be easily bound [70].

#### 6.2.2. Leaching

Large amounts of pesticides are registered and used worldwide, some of which are likely to leach to the groundwater and cause water pollution [31,71]. Leaching is influenced by several factors [31,45,46,47,48,49,50,51,52,53,54,55,56,57,58,59,60,61,62,63,64,65,66,67,68,69,70,71,72]. Singh (2002) indicates that solubility is an important factor for leaching because pesticides that are dissolved in water can move with the water in the soil. Soil permeability is another crucial factor influencing pesticide leaching [71]. Additionally, the greater the soil permeability, the higher the potential for pesticide leaching in the soil. The adsorption coefficient (Koc) and half-life in aerobic soil (DT50) are found to influence insecticide leaching [72]. Furthermore, the level of leaching also depends on how persistent the insecticide is in the environment. An insecticide low in persistence is less likely to leach because it may remain in the soil for a short time only [73]. For example, imidacloprid is persistent (DT50 in soil = 187 days), thus its environmental fate characteristics are high. Moreover, meteorological conditions, including annual rainfall and annual average temperature, are the main factors influencing the leaching characteristic of pesticides [31]. Precipitation is a key factor influencing the flux of downward leaching to groundwater together with insecticide solutes [74]. Furthermore, temperature impacts the evapotranspiration of soil which, in turn, influences the behavior of pesticide leaching in the soil. Soil properties such as soil texture and soil organic content affect water percolation and the transport of pesticides to groundwater [72,73,75]. Among these soil conditions, soil texture is the most important aspect that influences water movement and pesticide transport in the soil [76]. In addition, soil anaerobic microorganisms, organic matter content, and pH conditions are reported as important factors regulating the degradation of phenazines [73]. Boskovic et al. (2020) discussed the impact of soil properties on the absorption of pesticides. Their result indicated that the adsorption of both pesticides was highly correlated with pH (negatively correlated), and less associated with the soil organo-mineral complex (TOC, clay and surface area) and C and N in soil organic matter (OM). Particle sizes or cation exchange capacity (CEC) did not correlate with adsorption, but showed an association in multidimensional space in the factor analysis (FA). Moreover, the potential evaporation rate should be taken into account for the effect of crop residues on soil water and temperature regimes.

#### 6.2.3. Spray Drift

Spray drift is the airborne movement of spray droplets receding from a treatment site during pesticide application [76,77,78,79], thus causing environmental pollution and food contamination [31,72,80,81,82,83,84]. For example, aquatic ecosystems are the recipients of various pesticide residues, such as chlorpyrifos (ChF), due to leaching spray drift and agricultural runoff and cause toxicity in aquatic organisms, thus the oxidative stress enzymes and histological alterations in the vital organs of tilapia due to ChF exposure were investigated; the result of the study shows that sub-lethal concentrations of ChF can induce oxidative stress and histological alterations in the tissues of tilapia [85]. Another example is that although unmanned aerial vehicle (UAV) applications at low volume using fine and very fine droplets have been adopted in several commercial spray scenarios, allowing water-saving and high-efficiency operation in the delivery of pesticides, spray drift associated with UAV applications, especially for fine droplets generated from spinning disk nozzles, has not been fully discussed, which could raise the environmental and regulatory concerns. The drift potential of three different volume median diameters (VMDs or Dv0.5) of 100, 150, and 200 from a commercial quadcopter equipped with centrifugal nozzles exposed to different wind speeds under field conditions was compared. The results show that flight speed and altitude have a significant effect on the distribution of the airflow field [80].

#### 6.2.4. Volatilization

Volatilization is the conversion of a solid or a liquid into a gas. Once pesticides have been volatilized, they can be carried on air currents away from the treated surface [31]. Some important factors determine the volatilization level of the pesticide [72], including vapor pressure, temperature, humidity, air movement [86], and soil conditions such as texture, organic matter content, and moisture [87]. The higher the vapor pressure, the more volatile the insecticide will be. In addition, high temperatures, low relative humidity, and air movement tend to increase volatilization [72]. For example, there is more potential for the atmospheric dispersion of Organochlorine pesticides (OCPs) in tropical areas than in temperate climates [88]. Furthermore, an insecticide tightly adsorbed to soil particles is less likely to volatilize [89]. Lisouza et al. (2020) found that contaminated surface waters could be the major source of human exposure to OCPs through volatilization. Leaf, soil, and air samples were collected for 21 days after chlorpyrifos was applied to a field of purple tansy in order to further understand the fate and transport of the organophosphate insecticide, chlorpyrifos, and its degradation product, chlorpyrifos oxon. The result showed that the SCREEN3-predicted chlorpyrifos concentrations were >5 times higher than the measured concentrations. This indicates that approaches for calculating accurate pesticide volatilization fluxes from agricultural fields are still needed.

#### 6.2.5. Surface Runoff

Runoff is the movement of pesticides in water over a sloping surface [90]. Pesticides may move as compounds dissolved in water or attached to soil particles of the eroding soil. This has a close relationship with some factors, including the slope or grade of an area, edibility, texture and moisture content of the soil, amount and timing of rainfall, and irrigation [72].

Runoff is caused when the speed of water added to a field is so fast that it cannot be absorbed by the soil [31]. Over-irrigation results in the accumulation of excess surface water, causing insecticide runoff. Pesticide runoff results in pesticide pollution in streams, ponds, lakes, and wells, and pesticide contamination could negatively impact plants, animals, and humans [2,51].

## 7. Impact of Climate Change-Related Factors on Pesticide Use

**Figure 3:**
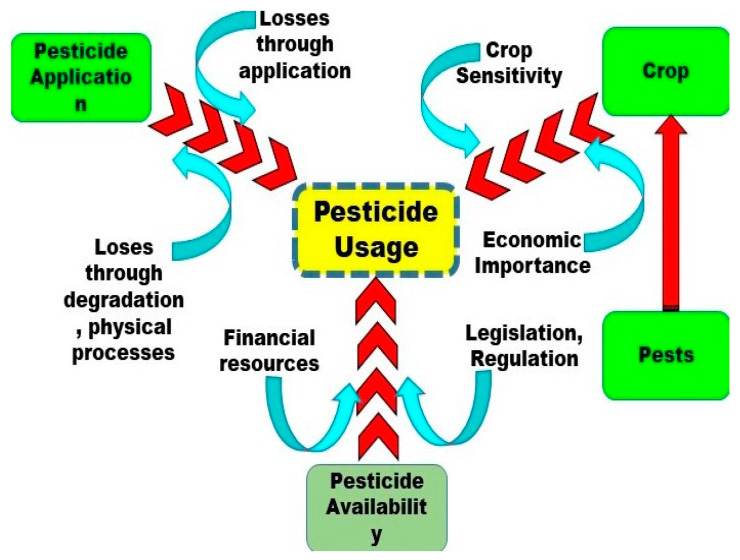
Influencing factors for pesticide use (by authors).

**Figure 4:**
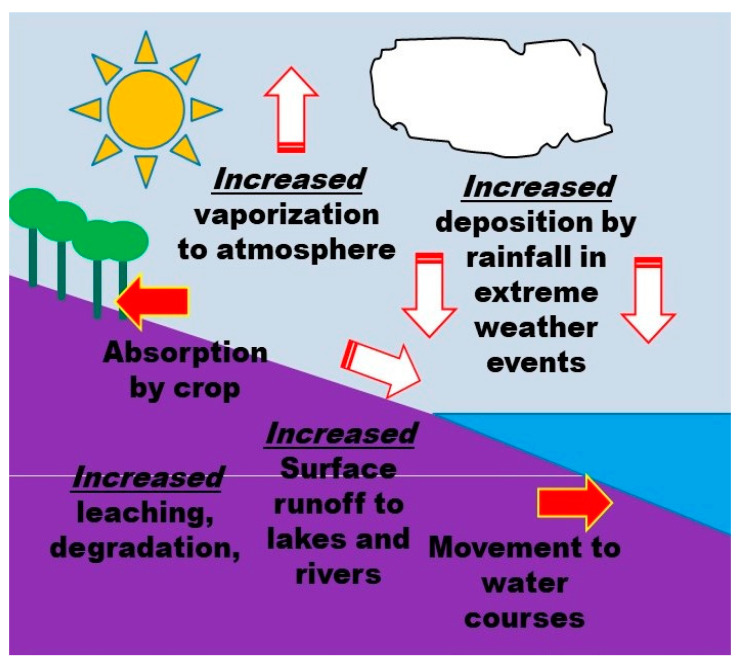
Climatic factors affect the environmental behavior of pesticides (by authors).

The use of synthetic pesticides has increased rapidly since World War II (1939–1945) to prevent, mitigate, or destroy pests, reduce agricultural production losses, and improve affordable yields and the quality of food [36]. Pesticide application is influenced by many factors, such as socioeconomic factors, environmental factors including soil condition, crop growth, and the occurrence of insect pests, weeds and diseases, and pesticide behavior in the environment (Figure 3). These factors are mostly influenced by climate change (Figure 4).

### 7.1. Influence on Soil Condition and Crop Growth

Climate change directly changes soil characteristics and leads to changes in pesticide applications [1]. Increased average temperature leads to a lower level of soil organic matter and results in enhanced potential for soil erosion with increased rates of the movement of water as well as organic and inorganic chemicals [91]. Moreover, owing to temperature increases, the capacity of soil to store and cycle carbon is altered [92], and the size and frequency of crack formation in soils will probably increase [38]. Bloomfield et al. (2006) further indicate that crack formation will lead to a more rapid and direct movement of water and solutes from the soil surface to a depth or directly to field drains, which could result in losses of pesticides in target areas and cause the pollution of ground and surface water.

The impact of climate change on soil conditions not only directly influences pesticide application but firstly impacts the distribution and growth of crops, insect pests, weeds, and diseases and then affects pesticide use. These indirect impacts will be discussed in the following section.

Climate factors influence the growth, survival, and reproduction of plants, limiting their geographic distribution, agricultural yield, and interaction with other species. Generally, food crops are either directly affected by changes in temperature, precipitation, and carbon dioxide or indirectly impacted by the effects of climate factors on soil, nutrient dynamics, and pest organisms [91].

Owing to the changes in mean and extreme temperatures and rainfall patterns, there will be positive and negative changes in crops grown in the different zones of cultivation, causing a change in the type and amount of pesticide application [91,93,94]. Specifically, weather changes such as erratic or low rainfall with a poor distribution may negatively impact crop performance and yields [95]. Abass et al. (2014) [96] argue that in some situations, where rainfall may not adequately support crop growth, planting seeds close to the onset of rainfall does not guarantee good yields, thus possibly causing a change in pesticide application. Additionally, drought conditions may cause a decline in both the growth and the special properties of crops. For example, Abass et al. (2014) point to the decline in growth and stimulant properties of tea crops under drought conditions. They also said that if the changes in tea functional quality due to poor water availability and herbivore pressures are indicative of broader climate change, on the one hand, tea production areas face increasingly extreme weather conditions predicting long-term and more frequent droughts, and increased heavy precipitation events on the other hand. This will affect the application of pesticides. Concerning higher temperatures, Ahmad et al. (2016) [97] point out that for sorghum production, the high temperatures in Pakistan from May to July are one of the major hurdles for this type of crop.

On the positive side, precipitation, temperature, and carbon dioxide levels rising to some extent during the growing seasons are always helpful to increase crop yields [98,99,100]. For example, on average, elevated carbon dioxide results in increased rice yields [100]. Furthermore, it is possible to produce dry-land green mealies in winter in areas with favorable summer rainfall and temperatures [98]. Climate change will also lengthen the active growing season, allowing an increase in farming, the introduction of new crops, and an opportunity for crop expansion, enabling the growth of new and additional crops, and possibly increase the use of pesticides [99].

### 7.2. Influence on Pests, Weeds, and Diseases

Crop damage caused by insects, pests, weeds, and diseases results from the complicated ecological dynamics of more than two organisms, making it very difficult to analyze and forecast [97]. Climate change could lead to changes in phenology and geographic distribution in a wide range of ecosystems [101]. The distribution and characteristics of pests, hosts, and bio-control agents having a relationship with the yield of crops are influenced by climate change [93,102]. Increased temperatures, changes in precipitation, and increased levels of carbon dioxide owing to climate change are the main factors affecting pest insects, weeds, and diseases [103,104,105,106,107].

#### 7.2.1. Pests in Crops

Regarding carbon dioxide, increases in atmospheric concentrations of CO_2_ may alter the susceptibility of many plants to herbivore insects because of the changes in plant nutrition and defense mechanisms [108,109]. Moreover, increases in CO_2_ would cause changes in the soil nitrogen content, the distribution of pests, and changes in population density [110]. Changes in precipitation will also cause changes in insect infestations. There are probably more severe insect infestations in wetter conditions [104,111]. Wetter conditions also change the geographical distributions of insects [112].

#### 7.2.2. Determinants of Diseases in Crops

Climate factors determine the type and distribution of crop diseases. Temperature, humidity, precipitation, radiation, and dew are the main climatic factors that impact crop diseases [92,94]. Climate conditions not only directly change the biological condition of plant hosts, pathogens, and vectors, and therefore influence crop diseases, they also directly impact the severity of diseases and plant losses [113,114,115,116,117,118,119,120,121,122].

Climate conditions, such as more frequent and abundant rainfall with increasing temperatures and higher concentrations of water vapor, will lead to favorable conditions for the development of infectious diseases and the germination of spores, as well as the activity and spread of the zoospores and their reproduction [123,124,125,126,127,128,129,130,131,132,133,134]. Moreover, increased winter rainfall may stimulate diseases [135]. Bloomfield et al. (2006) state that milder winters probably increase the survival of certain pests and diseases, increase their length of activity in a given year, and allow their establishment in new locations. Additionally, humidity and temperature variation will impact fungus growth, survival, and dissemination. Thus, climate change impacts the incidence of fungi and disease severity [91].

Vectors are also affected by climate change. Yi et al. (2014) [136] indicate that the lifecycle period of mosquitoes has been shortened by global warming, and it has also increased the transmission rates of diseases associated with mosquitoes. For example, there is a close negative relationship between the incidence of powdery mildew in sugar beet and the number of frost days in February and March [92].

Different disease responses to climate conditions can vary [137]. The study results by Kim et al. (2015) [138] show that pathogenic Escherichia coli had the strongest correlation with temperature and relative humidity, followed by Vibrio parahaemolyticus, Campylobacter jejuni, Salmonella spp., and Bacillus cereus. They also found that norovirus had a strong negative correlation with temperature and relative humidity, followed by Clostridium perfringens and Staphylococcus aureus that were poorly correlated with both temperature and relative humidity.

#### 7.2.3. Weed Growth

Climate change affects the growth of both crops and weeds [138]. Weeds are likely to evolve very rapidly in increasing levels of temperature, precipitation, and carbon dioxide, often resulting in a greater use of pesticides [139].

Increasing levels of carbon dioxide probably stimulate the growth and development of many weeds, causing some to become invasive [91,139,140,141]. Ziska et al. (2011) [140] indicate that increasing carbon dioxide levels probably increase the wind dispersal of weed seeds by increasing the height of the weed plant and plant size. This may cause farmers to use more or different herbicides. Increasing carbon dioxide impacts the growth of weeds and also leads to a decrease in the weeds’ herbicide efficacy. Moreover, increases in carbon dioxide promote morpho-physiological and anatomical changes in weeds, influencing the efficiency of the uptake and translocation of herbicides [142]. In addition, if vegetative growth is stimulated owing to increased photosynthesis in response to elevated CO_2_, perennial weeds may become even more noxious [143]. Furthermore, carbon dioxide promotes root biomass, which probably makes perennial weeds harder to control under higher carbon dioxide levels [144]. There is a decrease in stomata number and conductance but an increase in leaf thickness in C3 weeds with increasing carbon dioxide levels, which prevent the foliar uptake of herbicides [145].

Temperature is considered as one of the important aspects influencing the geographical distributions of weed species [141]. Temperature increases may change weed reproduction and weed competitive behavior. For example, the profuse growth of Datura stramonium L. needs high temperatures and probably becomes more competitive with increasing temperatures [146]. The survival and growth of winter annual weeds increase in milder and wetter winters, while the thermophile summer annuals probably grow larger in warmer summers with prolonged growing seasons [146]. Increased temperatures very strongly influence biomass accumulation by annual grass species during their reproductive phase [147]. A rising temperature not only affects the weed growth directly but also affects the uptake and translocation of herbicides in weeds and their persistence in soil, thus impacting the growth of the weeds [148]. Hence, farmers may be induced to try stronger herbicides to control weeds.

Weed growth and its interaction with crops are also influenced by variations in rainfall and drought conditions [149]. Due to the warming climate, increased extremely dry conditions and a variation of the rainfall pattern probably change weed distribution and also influence the weeds’ impact on crop production [150]. Moreover, different weeds respond to climate changes in different ways. For instance, C4 and parasitic weeds such as Striga hermonthica (probably thrive better under prolonged drought spells, Rhamphicarpa fistulosa (Hochst.) Benth is more suitable for excess water environments [142]. Additionally, increased rainfall frequency and intensity negatively impact the uptake, retention, and environmental behavior of herbicides [151]. Furthermore, droughts increase cuticle thickness and leaf pubescence, thus reducing herbicide entry into leaves [152]. Increased droughts also increase herbicide volatilization and unprecedented rises in rainfall could promote soil-applied herbicides and groundwater contamination [153]. Thus, precipitation increases could stimulate weed growth.

### 7.3. Influence on Pesticide Behavior in the Environment

Pesticide transformation and degradation as well as movement are the main pesticide behaviors in the environment [154,155]. Pesticide movement includes volatilization, runoff, and leaching processes, while pesticide degradation encompasses photolysis and chemical and microbial breakdown. These behaviors are influenced by climate change and climatic conditions (Figure 4).

#### 7.3.1. Volatilization

Volatilization is the main source of pesticide pollution in the atmosphere. Increasing temperatures, higher soil moisture, and direct exposure to sunlight result in rapid volatilization. For example, the concentration levels of currently used pesticides were higher during May and August due to intensive use and relatively high temperatures in the spring and summer in the Bo-Hai Sea atmosphere [1]. Another example is that the concentration levels of hexachlorocyclohexanes (HCHs) in the air are higher in summer than in winter in Hangzhou in the Yangtze River Delta region, China [156]. Similar to temperature, there is a rapid volatilization with humid soil after rainfall.

#### 7.3.2. Runoff and Drift

Runoff and drift are two main pathways to cause pesticide pollution in water [86]. The parcel’s slope, soil type, texture, and structure combined with crop growth, row directionality, and climatic factors are the main aspects that strongly affect the runoff rate [157]. Decoue et al. (2015) [93] also indicate that increased precipitation and higher temperatures exacerbate runoff contaminated with pesticides. The numbers and concentrations of pesticides have already been proven to rise spectacularly, sometimes resulting in a subsequent release into shallow groundwater [158,159].

#### 7.3.3. Leaching

The transfer of pesticides to a depth via leaching and to surface water via drainage was mostly influenced by interactions between climate and soil–pesticide combinations [93], enhancing the effect of precipitation volumes of variable duration, rainfall seasonality, intensity, and timing in relation with pesticide application [93]. Temperature affects soil mineralogy and geochemistry and is consequently the main cause for leaching [160]. In general, research describes a negative correlation with leaching often caused by desorption. Temperature not only causes a seasonal effect on pesticide transport in leaching but also reduces the influence of winter rainfall [91]. Such winter rain exhibits an overall strong influence on the more retained and less degraded residues of spring or autumn applications [93].

#### 7.3.4. Degradation

Global warming is acknowledged to accelerate the degradation of chemical components due to accelerated microbial and chemical reaction rates and may reduce concentrations of pesticides in the environment [91]. In addition, elevated soil moisture content and increased precipitation also enhance pesticide degradation and, accordingly, persistence [36]. Furthermore, a higher relative humidity was proven to induce faster environmental pesticide degradation, even though it is the more difficult initial degradation in this case [91].

## 8. Pesticide Contamination and Its Adverse Effects on the Natural Environment

Population growth and climate change contribute mainly to the increasing use of pesticides [161,162,163], and a higher global pesticide production is estimated in the future. Although pesticides play a significant role in improving crop yields and the production of affordable and good quality food, the increasing use of pesticides also brings a number of negative effects to the environment and human health [163]. Pesticides are used to kill pests and control weeds as a function of their chemical ingredients, therefore, they can also be toxic to other organisms, including birds, fish, beneficial insects, and non-target plants [25,164,165], as well as different environmental media, including air [166,167,168,169] water, soil, and crops [23,170]. Such chemical residues impact human health through the environment and food contamination. Moreover, pesticide contamination moves away from the target plants, resulting in environmental pollution. Pesticides move in several ways, including to the air, through wind currents, to water, through runoff or leaching, and to plants, animals, and humans [31,171].

### 8.1. Impact on Water

Many chemicals, including some pesticides, have been detected in surface water and groundwater [31,72]. It is widely accepted that pesticides enter both the surface water and groundwater by direct application for the control of aquatic weeds and aquatic insects, percolation and runoff from agricultural production fields, drift from agro-allied industrial wastewater, discharge from wastewater from clean-up equipment used for pesticide formulation and application [4,36,172] atmospheric deposition, and air/water exchange [173]. Groundwater is polluted when pesticides leach from the treated fields, mixing and washing sites, or waste disposal areas [174]. Surface water systems, including rivers, lakes, streams, reservoirs, and estuaries, are especially vulnerable to the accumulation of pesticides and other chemicals [172] since they are small captive sinks of the by-products of human activities. Surface water systems are linked to both groundwater and atmospheric water through the hydrologic cycle. Furthermore, pesticides in surface water can be transferred to groundwater through seepage of the soil. They also enter the atmosphere through evaporation and transpiration [175]. Atmospheric water and groundwater can also recharge surface waters.

Pesticide mobility in water results in pesticide contamination of water resources [31,176]. Both surface water and groundwater pollution caused by pesticides are very serious and urgent issues in freshwater and coastal ecosystems throughout the world [25,173,177]. Moreover, due to high costs and high technology requirements, it is difficult to treat polluted surface water, particularly polluted groundwater [25,178].

There are many reports about pesticide contamination of both surface water and groundwater worldwide [179,180,181,182,183,184]. For example, the United States Geological Survey (USGS) found several pesticides in more than 90% of water and fish samples collected from US streams [185]. Study results indicated that pesticide contamination had been reported in surface water in the Bohai Sea and the Yong-ding River of China, and the contamination levels of these areas varied in the different seasons. One report indicated that a higher concentration of glyphosate during dry seasons may be due to the reduced dilution from precipitation [186,187,188]. According to the OECD (2001) report, agriculture in the EU contributes 40–80% of total nitrogen and 20–40% of phosphorus to the pollution of surface waters. Another example is that herbicides have contaminated 37,000 to 500,000 m2 of the wetlands in Saskatchewan (Canada), and the contamination levels exceeded the national standard [176]. Such pesticide contamination in water not only directly impacts the drinking water quality in local areas but also causes indirect impacts by transferring to the next species, such as in soil and the food chain [189].

### 8.2. Impact on Soil

The capacity of soil to filter, degrade, and detoxify pesticides is a function or quality of soil [190]. The degradation of pesticides leads to the production of residues that persist and transform not only in aquatic ecosystems but also in terrestrial areas for years, posing a threat to the environment [191]. Indeed, soil and sediment contamination by pesticides has been a widespread problem in terrestrial areas that has caused adverse impacts on the quality of food and agricultural sustainability. Moreover, in terrestrial areas, because the soils reveal a large retention capacity of pesticides in their structures by adsorption but also re-emit old organic pollutants into the atmosphere, groundwater, and living organisms as a secondary source, soil is the principal reservoir of environmental pesticides, playing an important role in the global distribution and fate of contamination [67].

The persistence of pesticide residuals in the soil has a very close relationship with the properties of pesticides, including water solubility, soil sorption constant (Koc), the octanol/water partition coefficient (Kow), and half-life in soil (DT50) [192]. Pesticides that are strongly bound to soil have high Kow values resulting in high Koc values, and both properties result in strong sorption to the organic matter in the soil. Thus, pesticides classified as hydrophobic, persistent, and bio-accumulable would be expected to accumulate and persist in soils [25,193]. For example, some pesticides, such as organochlorine DDT, endosulfan, endrin, heptachlor, and lindane, are strongly bound to soil particles due to their persistency, thus they have been abandoned in many countries, including China [68]. Some other pesticides, including carbamates, fungicides, and some organophosphorus insecticides are not persistent in soil, but they may undergo different processes during runoff and leach into different environmental media. Therefore, soil contaminated by pesticides poses a widespread threat to water and the food chain.

The transformation behavior of pesticides in the soil is determined by the interactions between the soil matrixes, including organic matter content, pH, temperature, humidity, types of microorganism, irrigation modes, and grass hedges [36,68,157] and the pesticide properties, including the physical and chemical properties. For example, the larger the organic matter content, the greater the adsorption of pesticides. Additionally, adsorption increases with decreasing soil pH for 2, 4-D, 2, 4, 5-T, picloram, and atrazine pesticides [194].

### 8.3. Impact on Air

Pesticide contamination in the air is a considerable pollution factor that causes hazardous impacts on flora and fauna as well as human health [33]. The pesticides used for agricultural activities always spray drift in the air, and the residues of pesticides in the air are mainly from pesticide application or by volatilization from the soil or plants [195,196].

Pesticide sprays are one method of pesticide application. They are mainly projected by a fan as water droplets and, after a turbulent process when they go through the canopy, they are forced into the ground by gravity as well as drifted by atmospheric activities such as wind [70]. Subsurface application, surface application, and aerial spraying are three important methods of spraying widely used in the modern agricultural development process. However, hand spraying is still prevalent in many developing countries [197].

All methods of spraying pesticides have the potential to be inefficient and cause air pollution as well as to expose the general public to pesticides [25]. The pesticide residues are volatilized, dispersed, and transported over a long distance and are therefore subjected to a process of environmental recycling between the air and the terrestrial environment [198,199,200]. Pesticide drift could account for approximately 2% to 25% of pesticide loss during drifting [201]. This process not only produces pollution in the local environment but also brings adverse impacts to the global environment [23]. For example, pesticides including hexachlorocyclohexanes (HCHs), chlordane, and toxaphene were used in the fields in the south of the USA, where they were volatilized, transported by atmospheric processes, and then condensed in colder climates, and deposited from the atmosphere onto the Great Lakes in Canada [202]. Thus, it is difficult to assess the air pollution caused by pesticides.

### 8.4. Impact on Food Safety

The public and policymakers have raised a huge concern about toxic pesticides in food because of their negative health and environmental impacts. Food contamination is not only a consequence of spraying pesticides for non-target plants but also due to pesticide behavior in the environment, such as volatilization from the treated area to the air, soil, and non-target plants, and the residual pesticides transmitted from soil and water to crops, vegetables, and fruits [5,16,36]. This environmental behavior of pesticides and their residues lead to food contamination and damage to plants. For example, exposure to the herbicide clopyralid can reduce the yields of potato plants [203]. Volatilization of only 1% of the applied clopyralid is enough to damage non-target plants [25]. Aktar et al. (2009) also show that plants indirectly suffer from pesticide applications since pesticides are harmful to soil microorganisms and beneficial insects.

In some areas, pesticide residuals in crops and vegetables have exceeded the WHO maximum food contamination standards [25,69,204,205]. For example, Wanwimolruk et al. (2016) studied the pesticide contamination of fruits and vegetables and their health implications in Ghana, indicating that almost all of the studied fruits and vegetables contained pesticide residues above the maximum residue limits (MRLs). Lozowicka et al. (2015) [206] assessed the level of pesticide residues in vegetables in the Almaty region of Kazakhstan. They reported that more than half of the samples (59%) contained 29 pesticides, of which 10 were not registered in Kazakhstan, ranging from 0.01 to 0.88 mg kg^−1^, and 28% above the maximum residue levels (MRLs). Wanwimolruk et al. (2016) [206] showed that the detected pesticides exceeded their MRLs at a rate of 48% (local markets) and 35% (supermarkets) for Chinese kale and 71% (local markets) and 55% (supermarkets) for pak choi.

### 8.5. Impact on Non-Target Organisms

Unlike the targeted insect pests, non-target organisms are negatively influenced when pesticides are applied to the target plants [206]. This constitutes the damage to wildlife, birds, aquatic ecosystems, honeybees, and beneficial insects as well as the natural enemies of insect pests. Pesticides are detrimental to non-target organisms in two ways: firstly, the pesticides are harmful to non-target organisms through direct contact and secondly, the pesticide residuals may bring negative influences to non-target organisms at a later stage [25].

## 9. Conclusions and New Directions

Agricultural development has a long history throughout many locations around the world. The history of pesticide use during agricultural development can be divided into three periods of time. Pesticides are classified by different classification terms, such as chemical classes, functional groups, modes of action, and toxicity. Tremendous benefits have been achieved by using pesticides in a range of areas, including public health and agricultural activities. In terms of public health, pesticides are used in daily life to kill pests including mosquitoes, ticks, rats, and mice in houses, offices, malls, and streets. As a result, the immense burden of diseases caused by these vectors has been substantially reduced or eliminated. They have been used by farmers to control weeds and insects in agricultural cultivation, and remarkable increases in agricultural products have been reported as a result of pesticide use. When pesticides are used to target plants, pesticide behavior in the environment, such as transfer and degradation, should be considered. Improper pesticide usage and management and pesticide behavior in the environment lead to environmental pollution, including soil pollution, water pollution, air pollution, and food contamination.

Climate change factors influence socioeconomic factors and environmental factors including soil condition, crop growth, and the occurrence of insect pests, weeds, and diseases related to pesticide application and climate change factors also influence the pesticide behavior in the environment. (1) Soil conditions such as soil organic matter, the capacity of soil to store and cycle carbon, and the size and frequency of crack information in soils are influenced by climate change, leading to changes in pesticide application. (2) Climate change, including increased temperature, precipitation, and carbon dioxide, alters crop geographical distribution and affects crop productivity. Thus, climate change results in a potential rise in the volume and variety of pesticides. (3) Climate change affects crop growth, environmental conditions, the migration and distribution of insect pests, changes in the the abundance of pests, the numbers of pest outbreaks and the dissemination of vectors, the evolution of weeds, and the stimulation of diseases. These result in the increasing use of a wider variety of pesticides. (4) Climate change also accelerates the pesticide behavior of volatilization, runoff, leaching processes, and pesticide degradation that encompasses photolysis and chemical and microbial breakdown. Therefore, due to climate change, the increasing use of insecticides and pesticides increases exposure and human health risks from pesticide pollution.

Therefore, there is necessary to control pesticide contamination and its negative influence on environmental and other non-target organisms. Further studies should focus on both occupational and environmental exposures and their related health risk assessment of pesticides to better understand pesticide use and management in the future. To minimize the negative influence of pesticide contamination on the environment and non-target organisms, new scientific methodology and technology and useful measures, such as integrated pest management (IPM), laws that forbid pesticides with high risks, and the development of a national implementation plan (NIP), should be implemented, to reduce the negative effects of pesticides. Furthermore, it is crucial to convey the scientific outcomes of the exposure and occupational and environmental health risk assessments to provide scientific training for pesticide application, the prevention of adverse health effects of pesticide usage, and the promotion of safety for applicators and communities to support sustainable development. Biopesticides should also be developed alongside chemical pesticides to minimize pesticide contamination.

## References

[1] Bernardes M.F.F., Pazin M., Pereira L.C., Dorta D.J. (2015). Impact of Pesticides on Environmental and Human Health. Toxicology Studies—Cells, Drugs and Environment.

[2] Aktar W., Paramasivam M., Sengupta D., Purkait S., Ganguly M., Banerjee S. (2008). Impact assessment of pesticide residues in fish of Ganga river around Kolkata in West Bengal. Environ. Monit. Assess..

[3] Fenik J., Tankiewicz M., Biziuk M. (2011). Properties and determination of pesticides in fruits and vegetables. TrAC Trends Anal. Chem..

[4] Strassemeyer J., Daehmlow D., Dominic A., Lorenz S., Golla B. (2017). SYNOPS-WEB, an online tool for environmental risk assessment to evaluate pesticide strategies on field level. Crop. Prot..

[5] Carvalho F.P. (2017). Pesticides, environment, and food safety. Food Energy Secur..

[6] Hayes T.B., Hansen M., Kapuscinski A.R., Locke K.A., Barnosky A. (2017). From silent spring to silent night: Agrochemicals and the anthropocene. Elem Sci Anth.

[7] Hernández A.F., Gil F., Lacasaña M., Rodríguez-Barranco M., Tsatsakis A.M., Requena M., Alarcón R. (2013). Pesticide exposure and genetic variation in xenobiotic-metabolizing enzymes interact to induce biochemical liver damage. Food Chem. Toxicol..

[8] Unsworth J. (2010). History of Pesticide Use. http://agrochemicals.iupac.org/index.php?option=com_sobi2&sobi2Task=sobi2Details&catid=3&sobi2Id=31.

[9] Kislev M.E., Weiss E., Hartmann A. (2004). Impetus for sowing and the beginning of agriculture: Ground collecting of wild cereals. Proc. Natl. Acad. Sci. USA.

[10] Davies O. (1968). The Origins of Agriculture in West Africa. Curr. Anthr..

[11] Lu T.L.D. (1999). The Transition from Foraging to Farming and the Origin of Agriculture in China, British Archaeological Reports Limited. https://www.google.com.au/books/edition/The_Transition_from_Foraging_to_Farming/_tkLAAAAYAAJ?hl=en.

[12] Wilkes H.G. (1979). Mexico and Central America as a Centre for the Origin of Agriculture and the Evolution of Maize. Crop Improvement.

[13] Zhang W., Jiang F., Ou J. (2011). Global pesticide consumption and pollution: With China as a focus. Proc. Int. Acad. Ecol. Environ. Sci..

[14] Council N.R. (2000). The Future Role of Pesticides in US Agriculture.

[15] Sheail J. (1991). The regulation of pesticides use: An historical perspective. Innovation and Environmental Risks.

[16] Zhang Q., Xia Z., Wu M., Wang L., Yang H. (2017). Human health risk assessment of DDTs and HCHs through dietary exposure in Nanjing, China. Chemosphere.

[17] Ross G. (2005). Risks and benefits of DDT. Lancet.

[18] Zhang K., Zhang B.-Z., Li S.-M., Zeng E.Y. (2011). Regional dynamics of persistent organic pollutants (POPs) in the Pearl River Delta, China: Implications and perspectives. Environ. Pollut..

[19] Barnhoorn I.E.J., Bornman M., Van Rensburg C.J., Bouwman H. (2009). DDT residues in water, sediment, domestic and indigenous biota from a currently DDT-sprayed area. Chemosphere.

[20] Garcia F.P., Ascencio S.Y.C., Oyarzun J.C.G., Hernandez A.C., Alavarado P.V. (2012). Pesticides: Classification, uses and toxicity. Measures of exposure and genotoxic risks. Int. J. Environ. Sci. Toxic. Res..

[21] Amaral A.F.S. (2014). Pesticides and Asthma: Challenges for Epidemiology. Front. Public Health.

[22] Mnif W., Hassine A.I.H., Bouaziz A., Bartegi A., Thomas O., Roig B. (2011). Effect of Endocrine Disruptor Pesticides: A Review. Int. J. Environ. Res. Public Health.

[23] Kim K.-H., Kabir E., Jahan S.A. (2017). Exposure to pesticides and the associated human health effects. Sci. Total Environ..

[24] Zhang C., Sun Y., Hu R., Huang J., Huang X., Li Y., Yin Y., Chen Z. (2018). A comparison of the effects of agricultural pesticide uses on peripheral nerve conduction in China. Sci. Rep..

[25] Aktar W., Sengupta D., Chowdhury A. (2009). Impact of pesticides use in agriculture: Their benefits and hazards. Interdiscip. Toxicol..

[26] Lawler S.P. (2017). Environmental safety review of methoprene and bacterially-derived pesticides commonly used for sustained mosquito control. Ecotoxicol. Environ. Saf..

[27] Lamichhane J.R. (2017). Pesticide use and risk reduction in European farming systems with IPM: An introduction to the special issue. Crop. Prot..

[28] Cooper J., Dobson H. (2007). The benefits of pesticides to mankind and the environment. Crop. Prot..

[29] Ridsdill-Smith T., Pavri C. (2000). Single Spring Spray Protects Pastures. https://www.agric.wa.gov.au/pastures/spray-topping-declared-plants.

[30] Miller S.F. (1982). The effects of weed control technological change on rural communities. Outlook Agric..

[31] Singh D.K. (2012). Pesticides and Environment. Pestic. Chem. Toxicol..

[32] Scholtz M., Bidleman T.F. (2007). Modelling of the long-term fate of pesticide residues in agricultural soils and their surface exchange with the atmosphere: Part II. Projected long-term fate of pesticide residues. Sci. Total Environ..

[33] Liu Y., Mo R., Tang F., Fu Y., Guo Y. (2015). Influence of different formulations on chlorpyrifos behavior and risk assessment in bamboo forest of China. Environ. Sci. Pollut. Res..

[34] Marie L., Payraudeau S., Benoit G., Maurice M., Gwenaël I. (2017). Degradation and Transport of the Chiral Herbicide S-Metolachlor at the Catchment Scale: Combining Observation Scales and Analytical Approaches. Environ. Sci. Technol..

[35] Robinson D.E., Mansingh A., Dasgupta T.P. (1999). Fate and transport of ethoprophos in the Jamaican environment. Sci. Total Environ..

[36] Damalas C.A., Eleftherohorinos I. (2011). Pesticide Exposure, Safety Issues, and Risk Assessment Indicators. Int. J. Environ. Res. Public Health.

[37] Abian J., Durand G., Barceló J. (1993). Analysis of chlorotriazines and their degradation products in environmental samples by selecting various operating modes in thermospray HPLC/MS/MS. J. Agric. Food Chem..

[38] Wu L.P., Chládková B., Lechtenfeld O.J., Lian S., Schindelka J., Herrmann H., Richnow H.H. (2018). Characterizing chemical transformation of organophosphorus compounds by 13C and 2H stable isotope analysis. Sci. Total Environ..

[39] Tcaciuc A.P., Borrelli R., Zaninetta L.M., Gschwend P.M., Tcaciuc P. (2018). Passive sampling of DDT, DDE and DDD in sediments: Accounting for degradation processes with reaction–diffusion modeling. Environ. Sci. Process. Impacts.

[40] Tariq S.R., Nisar L. (2018). Reductive transformation of profenofos with nanoscale Fe/Ni particles. Environ. Monit. Assess..

[41] Zhao Y., Wendling L.A., Wang C., Pei Y. (2016). Behavior of chlorpyrifos and its major metabolite TCP (3,5,6-trichloro-2-pyridinol) in agricultural soils amended with drinking water treatment residuals. J. Soils Sediments.

[42] Yue L., Ge C., Feng D., Yu H., Deng H., Fu B. (2017). Adsorption–desorption behavior of atrazine on agricultural soils in China. J. Environ. Sci..

[43] Luo C., Huang Y., Huang D., Liu M., Xiong W., Guo Q., Yang T. (2018). Migration and Transformation Characteristics of Niclosamide in a Soil–Plant System. ACS Omega.

[44] Su W., Hao H., Xu H., Lu C., Wu R., Xue F. (2016). Degradation of Mesotrione Affected by Environmental Conditions. Bull. Environ. Contam. Toxicol..

[45] Han D.M., Tong X.X., Jin M.G., Hepburn E., Tong C.S., Song X.F. (2012). Evaluation of organic contamination in urban groundwater surrounding a municipal landfill, Zhoukou, China. Environ. Monit. Assess..

[46] Qian S., Zhu H., Xiong B., Zheng G., Zhang J., Xu W. (2017). Adsorption and desorption characteristics of endosulfan in two typical agricultural soils in Southwest China. Environ. Sci. Pollut. Res..

[47] Qian J., Li J., Fang D., Yu Y., Zhi J. (2014). A disposable biofilm-modified amperometric biosensor for the sensitive determination of pesticide biotoxicity in water. RSC Adv..

[48] Bansal O.P. (2011). Fate of pesticides in the environment. J. Indian Chem. Soc..

[49] Quan G., Yin C., Chen T., Yan J. (2015). Degradation of Herbicide Mesotrione in Three Soils with Differing Physicochemical Properties from China. J. Environ. Qual..

[50] Wei J., Chen Y., Tiemur A., Wang J.-D., Wu B. (2018). Degradation of pesticide residues by gaseous chlorine dioxide on table grapes. Postharvest Biol. Technol..

[51] Qin F., Gao Y.X., Guo B.Y., Xu P., Li J.Z., Wang H. (2014). Environmental behavior of benalaxyl and furalaxyl enantiomers in agricultural soils. J. Environ. Sci. Health Part B.

[52] Xue N., Yang R., Xu X., Seip H.M., Zang Q., Zeng Q. (2006). Adsorption and Degradation of Benfuracarb in Three Soils in Hunan, People’s Republic of China. Bull. Environ. Contam. Toxicol..

[53] Liu Y., Xu Z., Wu X., Gui W., Zhu G. (2010). Adsorption and desorption behavior of herbicide diuron on various Chinese cultivated soils. J. Hazard. Mater..

[54] Alvarez D.O., Mendes K.F., Tosi M., De Souza L.F., Cedano J.C.C., Falcão N.P.D.S., Dunfield K., Tsai S.M., Tornisielo V.L. (2021). Sorption-desorption and biodegradation of sulfometuron-methyl and its effects on the bacterial communities in Amazonian soils amended with aged biochar. Ecotoxicol. Environ. Saf..

[55] Bošković N., Brandstätter-Scherr K., Sedláček P., Bílková Z., Bielská L., Hofman J. (2020). Adsorption of epoxiconazole and tebuconazole in twenty different agricultural soils in relation to their properties. Chemosphere.

[56] Liu J., Zhou J.H., Guo Q.N., Ma L.Y., Yang H. (2021). Physiochemical assessment of environmental behaviors of herbicide atrazine in soils associated with its degradation and bioavailability to weeds. Chemosphere.

[57] Dong D.M., Lan X.H., Guo Z.Y., HUA X.Y. (2013). Sorption Kinetics of Organochlorine Pesticides on Three Types of Solids in Natural Waters. Chem. J. Chin. Univ.-Chin..

[58] Ren W., Wang M., Zhou Q. (2011). Effect of soil pH and organic matter on desorption hysteresis of chlorimuron-ethyl in two typical Chinese soils. J. Soils Sediments.

[59] Si Y., Zhang J., Wang S., Zhang L., Zhou D. (2006). Influence of organic amendment on the adsorption and leaching of ethametsulfuron-methyl in acidic soils in China. Geoderma.

[60] Yuan G., Qin J.-X., Li J., Lang X.-X., Wang G.H. (2014). Persistent organic pollutants in soil near the Changwengluozha glacier of the Central Tibetan Plateau, China: Their sorption to clays and implication. Sci. Total Environ..

[61] Wu D., Yun Y., Jiang L., Wu C. (2018). Influence of dissolved organic matter on sorption and desorption of MCPA in ferralsol. Sci. Total Environ..

[62] Wu P., Wu W.Z., Han Z.H., Yang H. (2016). Desorption and mobilization of three strobilurin fungicides in three types of soil. Environ. Monit. Assess..

[63] Duan L., Wang W., Sun Y. (2013). Ammonium Nitrogen Adsorption-Desorption Characteristics and Its Hysteresis of Typical Soils from Guanzhong Basin, China. Asian J. Chem..

[64] Gao C., Yang S., Wang W., Gao L. (2014). Influence of Humic Acid Colloid on Adsorption of DDT in the Riverbed Sediments. Asian J. Chem..

[65] Gao F., Jia J., Wang X. (2008). Occurrence and Ordination of Dichlorodiphenyltrichloroethane and Hexachlorocyclohexane in Agricultural Soils from Guangzhou, China. Arch. Environ. Contam. Toxicol..

[66] Duan L., Zhang N., Wang Y., Zhang C.D., Zhu L.Y., Chen W. (2008). Release of hexachlorocyclohexanes from historically and freshly contaminated soils in China: Implications for fate and regulation. Environ. Pollut..

[67] Al-Wabel M.I., El-Saeid M.H., El-Naggar A.H., Alromian F., Osman K., Elnazi K., Sallam A.S. (2016). Spatial distribution of pesticide residues in the groundwater of a condensed agricultural area. Arab. J. Geosci..

[68] Yadav I.C., Devi N.L., Syed J.H., Cheng Z., Li J., Zhang G., Jones K.C. (2015). Current status of persistent organic pesticides residues in air, water, and soil, and their possible effect on neighboring countries: A comprehensive review of India. Sci. Total Environ..

[69] Lozowicka B., Abzeitova E., Sagitov A., Kaczyński P., Toleubayev K., Li A. (2015). Studies of pesticide residues in tomatoes and cucumbers from Kazakhstan and the associated health risks. Environ. Monit. Assess..

[70] Ðurišić-Mladenović N., Škrbić B., Cvejanov J. (2010). Organochlorine Pesticides in Soil and Sediment from an Urban Zone of Novi Sad, Serbia. Survival and Sustainability: Environmental Concerns in the 21st Century.

[71] Fontana A., Lana N.B., Martínez L.D., Altamirano J.C. (2010). Ultrasound-assisted leaching-dispersive solid-phase extraction followed by liquid–liquid microextraction for the determination of polybrominated diphenyl ethers in sediment samples by gas chromatography–tandem mass spectrometry. Talanta.

[72] Connell D.W. (2005). Basic Concepts of Environmental Chemistry.

[73] Geng Y., Ma J., Zhou R., Jia R., Li C. (2017). Assessment of insecticide risk to human health in groundwater in Northern China by using the China-PEARL model. Pest Manag. Sci..

[74] Labite H., Holden N.M., Richards K.G., Kramers G., Premrov A., Coxon C., Cummins E. (2013). Comparison of pesticide leaching potential to groundwater under EU FOCUS and site specific conditions. Sci. Total Environ..

[75] Sijm D., Rikken M., Rorije E., Traas T., McLachlan M., Peijnenburg W. (2007). Transport, Accumulation and Transformation Processes.

[76] Ou J., Li H., Ou X., Yang Z., Chen M., Liu K., Teng Y., Xing B. (2020). Degradation, adsorption and leaching of phenazine-1-carboxamide in agricultural soils. Ecotoxicol. Environ. Saf..

[77] Grella M., Miranda-Fuentes A., Marucco P., Balsari P., Gioelli F. (2020). Development of Drift-Reducing Spouts For Vineyard Pneumatic Sprayers: Measurement of Droplet Size Spectra Generated and Their Classification. Appl. Sci..

[78] Pourreza A., Moghimi A., Niederholzer F.J.A., Larbi P.A., Zuniga-Ramirez G., Cheung K.H., Khorsandi F. (2020). Spray Backstop: A Method to Reduce Orchard Spray Drift Potential without Limiting the Spray and Air Delivery. Sustainability.

[79] Wang G., Han Y., Li X., Andaloro J., Chen P., Hoffmann W.C., Han X., Chen S., Lan Y. (2020). Field evaluation of spray drift and environmental impact using an agricultural unmanned aerial vehicle (UAV) sprayer. Sci. Total Environ..

[80] Zhang H., Qi L., Wu Y., Musiu E.M., Cheng Z., Wang P. (2020). Numerical simulation of airflow field from a six–rotor plant protection drone using lattice Boltzmann method. Biosyst. Eng..

[81] Van Steenwyk R.A., Siegel J.P., Bisabri B., Cabuslay C.S., Choi J.M., Steggall J., Mace K.C., Blecker S.W., Poe P.A., Peters-Collaer S.R. (2020). Spray drift mitigation using opposing synchronized air-blast sprayers. Pest Manag. Sci..

[82] Grella M., Marucco P., Balafoutis A., Balsari P. (2020). Spray Drift Generated in Vineyard during Under-Row Weed Control and Suckering: Evaluation of Direct and Indirect Drift-Reducing Techniques. Sustainability.

[83] Vieira B.C., Butts T.R., Rodrigues A.O., Schleier J.J., Fritz B.K., Kruger G. (2020). Particle drift potential of glyphosate plus 2,4-D choline pre-mixture formulation in a low-speed wind tunnel. Weed Technol..

[84] Ghaste M., Hayden N.C., Osterholt M.J., Young J., Young B., Widhalm J.R. (2020). Evaluation of a Stable Isotope-Based Direct Quantification Method for Dicamba Analysis from Air and Water Using Single-Quadrupole LC–MS. Molecules.

[85] Farhan M., Wajid A., Hussain T., Jabeen F., Ishaque U., Iftikhar M., Daim M.A., Noureen A. (2020). Investigation of oxidative stress enzymes and histological alterations in tilapia exposed to chlorpyrifos. Environ. Sci. Pollut. Res..

[86] Zhu S., Niu L., Aamir M., Zhou Y., Xu C., Liu W. (2017). Spatial and seasonal variations in air-soil exchange, enantiomeric signatures and associated health risks of hexachlorocyclohexanes (HCHs) in a megacity Hangzhou in the Yangtze River Delta region, China. Sci. Total Environ..

[87] Alamdar A., Syed J.H., Malik R.N., Katsoyiannis A., Liu J., Li J., Zhang G., Jones K.C. (2014). Organochlorine pesticides in surface soils from obsolete pesticide dumping ground in Hyderabad City, Pakistan: Contamination levels and their potential for air–soil exchange. Sci. Total Environ..

[88] Chakraborty P., Zhang G., Li J., Sivakumar A., Jones K.C. (2015). Occurrence and sources of selected organochlorine pesticides in the soil of seven major Indian cities: Assessment of air–soil exchange. Environ. Pollut..

[89] Wong F., Alegria H.A., Jantunen L.M., Bidleman T.F., Salvador-Figueroa M., Gold-Bouchot G., Ceja-Moreno V., Waliszewski S.M., Infanzon R. (2008). Organochlorine pesticides in soils and air of southern Mexico: Chemical profiles and potential for soil emissions. Atmos. Environ..

[90] Das S., Hageman K.J., Taylor M., Michelsen-Heath S., Stewart I. (2020). Fate of the organophosphate insecticide, chlorpyrifos, in leaves, soil, and air following application. Chemosphere.

[91] Bloomfield J.P., Williams R., Gooddy D., Cape J., Guha P. (2006). Impacts of climate change on the fate and behaviour of pesticides in surface and groundwater—A UK perspective. Sci. Total Environ..

[92] Sistla S.A., Moore J.C., Simpson R.T., Gough L., Shaver G.R., Schimel J.P. (2013). Long-term warming restructures Arctic tundra without changing net soil carbon storage. Nat. Cell Biol..

[93] Delcour I., Spanoghe P., Uyttendaele M. (2015). Literature review: Impact of climate change on pesticide use. Food Res. Int..

[94] Lennon J.J. (2015). Potential impacts of climate change on agriculture and food safety within the island of Ireland††This paper is one of a series of reviews on “Climate Change and Food Safety—An Island of Ireland perspective”. Trends Food Sci. Technol..

[95] Marvin H.J., Kleter G.A., Van Der Fels-Klerx H., Noordam M.Y., Franz E., Willems D.J., Boxall A. (2013). Proactive systems for early warning of potential impacts of natural disasters on food safety: Climate-change-induced extreme events as case in point. Food Control..

[96] Abass A.B., Ndunguru G., Mamiro P., Alenkhe B., Mlingi N., Bekunda M. (2014). Post-harvest food losses in a maize-based farming system of semi-arid savannah area of Tanzania. J. Stored Prod. Res..

[97] Ahmad W., Noor M.A., Afzal I., Bakhtavar M.A., Nawaz M.M., Sun X., Zhou B., Ma W., Zhao M. (2016). Improvement of Sorghum Crop through Exogenous Application of Natural Growth-Promoting Substances under a Changing Climate. Sustainability.

[98] Ainsworth E.A. (2008). Rice production in a changing climate: A meta-analysis of responses to elevated carbon dioxide and elevated ozone concentration. Glob. Chang. Biol..

[99] Akinnuoye-Adelabu D.B., Modi A.T., Mabhaudhi T. (2017). Potential of producing green mealies in summer and winter at two sites in KwaZulu-Natal, South Africa, considering rainfall, soil moisture and weeding. S. Afr. J. Plant Soil.

[100] Noyes P.D., McElwee M.K., Miller H.D., Clark B.W., Van Tiem L.A., Walcott K.C., Erwin K.N., Levin E.D. (2009). The toxicology of climate change: Environmental contaminants in a warming world. Environ. Int..

[101] Scherm H. (2004). Climate change: Can we predict the impacts on plant pathology and pest management?. Can. J. Plant Pathol..

[102] Meynard C.N., Gay P.-E., Lecoq M., Foucart A., Piou C., Chapuis M.-P. (2017). Climate-driven geographic distribution of the desert locust during recession periods: Subspecies’ niche differentiation and relative risks under scenarios of climate change. Glob. Chang. Biol..

[103] Fontaine J.J., Decker K.L., Skagen S.K., Van Riper C. (2009). Spatial and temporal variation in climate change: A bird’s eye view. Clim. Chang..

[104] Jones L.M., Koehler A.K., Trnka M., Balek J., Challinor A.J., Atkinson H.J., Urwin P.E. (2017). Climate change is predicted to alter the current pest status of Globodera pallida and G. rostochiensis in the United Kingdom. Glob. Chang. Biol..

[105] Lesk C., Coffel E., D’Amato A.W., Dodds K., Horton R. (2017). Threats to North American forests from southern pine beetle with warming winters. Nat. Clim. Chang..

[106] López-Blanco E., Lund M., Williams M., Tamstorf M.P., Westergaard-Nielsen A., Exbrayat J.-F., Hansen B.U., Christensen T.R. (2017). Exchange of CO_2_ in Arctic tundra: Impacts of meteorological variations and biological disturbance. Biogeosciences.

[107] Olfert O., Weiss R., Elliott R., Soroka J. (2017). Bioclimatic approach to assessing the potential impact of climate change on two flea beetle (Coleoptera: Chrysomelidae) species in Canada. Can. Èntomol..

[108] Sultana S., Baumgartner J.B., Dominiak B.C., Royer J.E., Beaumont L.J. (2017). Potential impacts of climate change on habitat suitability for the Queensland fruit fly. Sci. Rep..

[109] Guo H., Wan S., Ge F. (2017). Effect of elevated CO_2_ and O_3_ on phytohormone-mediated plant resistance to vector insects and insect-borne plant viruses. Sci. China Life Sci..

[110] Frew A., Allsopp P.G., Gherlenda A.N., Johnson S.N. (2016). Increased root herbivory under elevated atmospheric carbon dioxide concentrations is reversed by silicon-based plant defences. J. Appl. Ecol..

[111] Patterson D.T., Westbrook J.K., Joyce† R., Lingren P.D., Rogasik J. (1999). Weeds, Insects, and Diseases. Clim. Chang..

[112] Rosenzweig C., Iglesias A., Yang X., Epstein P.R., Chivian E. (2001). Climate Change and Extreme Weather Events; Implications for Food Production, Plant Diseases, and Pests. Glob. Chang. Hum. Health.

[113] Caubel J., Launay M., Ripoche D., Gouache D., Buis S., Huard F., Huber L., Brun F., Bancal M.O. (2017). Climate change effects on leaf rust of wheat: Implementing a coupled crop-disease model in a French regional application. Eur. J. Agron..

[114] Garrett K.A., Andersen K.F., Asche F., Bowden R.L., Forbes G.A., Kulakow P.A., Zhou B. (2017). Resistance Genes in Global Crop Breeding Networks. Phytopathology.

[115] Runion G.B. (2003). Climate change and plant pathosystems—future disease prevention starts here. New Phytol..

[116] Rydzanicz K., Kiewra D., Lonc E. (2006). Changes in range of mosquito-borne diseases affected by global climatic fluctuations. Wiad. Parazytol..

[117] Savary S., Djurle A., Yuen J., Ficke A., Rossi V., Esker P.D., Fernandes J.M.C., Del Ponte E.M., Kumar J., Madden L.V. (2017). A White Paper on Global Wheat Health Based on Scenario Development and Analysis. Phytopathology.

[118] Singhvi N., Kushwaha R., Mathur S., Suranarayana N. (2007). Role of weather factors on disease and pest incidence on tasar food plants and silkworms and developing operational disease and pest forewarning system for tasar sericulture. J. Exp. Zool..

[119] Tubby K.V., Webber J.F. (2010). Pests and diseases threatening urban trees under a changing climate. Forestry.

[120] Viswanathan R. (2013). Sustainable ecofriendly disease management systems in sugarcane production under the changing climate. J. Mycol. Plant Pathol..

[121] Wei J., Hansen A., Zhang Y., Li H., Liu Q., Sun Y., Xue S., Zhao S., Bi P. (2014). The Impact of Climate Change on Infectious Disease Transmission: Perceptions of CDC Health Professionals in Shanxi Province, China. PLoS ONE.

[122] Yacoub S., Kotit S., Yacoub M.H. (2011). Disease appearance and evolution against a background of climate change and reduced resources. Philos. Trans. R. Soc. A Math. Phys. Eng. Sci..

[123] Yamamura K., Yokozawa M. (2002). Prediction of a geographical shift in the prevalence of rice stripe virus disease transmitted by the small brown planthopper, Laodelphax striatellus(Fallen)(Hemiptera: Delphacidae), under global warming. Appl. Entomol. Zool..

[124] Anderson P.K., Cunningham A.A., Patel N.G., Morales F.J., Epstein P.R., Daszak P. (2004). Emerging infectious diseases of plants: Pathogen pollution, climate change and agrotechnology drivers. Trends Ecol. Evol..

[125] Boland G., Melzer M., Hopkin A., Higgins V., Nassuth A. (2004). Climate change and plant diseases in Ontario. Can. J. Plant Pathol..

[126] Boyd I.L., Freer-Smith P.H., Gilligan C.A., Godfray H.C.J. (2013). The Consequence of Tree Pests and Diseases for Ecosystem Services. Science.

[127] Branco M., Bragança H., Sousa E., Phillips A.J. (2014). Pests and Diseases in Portuguese Forestry: Current and New Threats. World For..

[128] Carisse O. (2017). Using biovigilance-based information for strategic and tactical disease management decisions. Can. J. Plant Pathol..

[129] Coakley S. (2012). Projected effects of climate change on plant disease and how plant pathologists can prepare to meet the challenge. Can. J. Plant Pathol.-Rev. Can. Phytopathol..

[130] Colosio C. (2013). Agriculture in Italy nowadays: Ancient risks and emerging diseases. G. Ital. Med. Lav. Ergon..

[131] Davies S., Patenaude G., Snowdon P. (2017). A new approach to assessing the risk to woodland from pest and diseases. Forestry.

[132] Dereure J., Vanwambeke S.O., Malé P., Martínez S., Pratlong F., Balard Y., Dedet J.-P. (2009). The Potential Effects of Global Warming on Changes in Canine Leishmaniasis in a Focus outside the Classical Area of the Disease in Southern France. Vector-Borne Zoonotic Dis..

[133] Dietrich J.P., Van Gaest A.L., Strickland S.A., Arkoosh M.R. (2014). The impact of temperature stress and pesticide exposure on mortality and disease susceptibility of endangered Pacific salmon. Chemosphere.

[134] Evans N., Butterworth M.H., Baierl A., Semenov M.A., West J.S., Barnes A., Moran D., Fitt B.D.L. (2010). The impact of climate change on disease constraints on production of oilseed rape. Food Secur..

[135] Garrett K., Dobson A., Kroschel J., Natarajan B., Orlandini S., Tonnang H., Valdivia C. (2013). The effects of climate variability and the color of weather time series on agricultural diseases and pests, and on decisions for their management. Agric. For. Meteorol..

[136] Yi H., Devkota B.R., Yu J.-S., Oh K.-C., Kim J., Kim H.-J. (2014). Effects of global warming on mosquitoes & mosquito-borne diseases and the new strategies for mosquito control. Èntomol. Res..

[137] Al-Askar A.A., Ghoneem K.M., Rashad Y.M., Abdulkhair W.M., Hafez E.E., Shabana Y.M., Baka Z.A. (2014). Occurrence and distribution of tomato seed-borne mycoflora in S audi A rabia and its correlation with the climatic variables. Microb. Biotechnol..

[138] Kim Y.S., Park K.H., Chun H.S., Choi C., Bahk G.J. (2015). Correlations between climatic conditions and foodborne disease. Food Res. Int..

[139] Ziska L.H., Goins E.W. (2006). Elevated Atmospheric Carbon Dioxide and Weed Populations in Glyphosate Treated Soybean. Crop. Sci..

[140] Ziska L.H., Blumenthal D.M., Runion G.B., Hunt E.R., Diaz-Soltero H. (2010). Invasive species and climate change: An agronomic perspective. Clim. Chang..

[141] Ziska L.H., Faulkner S., Lydon J. (2004). Changes in biomass and root:shoot ratio of field-grown Canada thistle (Cirsium arvense), a noxious, invasive weed, with elevated CO2: Implications for control with glyphosate. Weed Sci..

[142] Ramesh K., Matloob A., Aslam F., Florentine S., Chauhan B.S. (2017). Weeds in a Changing Climate: Vulnerabilities, Consequences, and Implications for Future Weed Management. Front. Plant Sci..

[143] Ziska L.H., Teasdale J.R. (2000). Sustained growth and increased tolerance to glyphosate observed in a C3 perennial weed, quackgrass (Elytrigia repens), grown at elevated carbon dioxide. Funct. Plant Biol..

[144] Manea A., Leishman M.R., Downey P.O. (2011). Exotic C4Grasses Have Increased Tolerance to Glyphosate under Elevated Carbon Dioxide. Weed Sci..

[145] Nowak K.M., Girardi C., Miltner A., Gehre M., Schäffer A., Kästner M. (2013). Contribution of microorganisms to non-extractable residue formation during biodegradation of ibuprofen in soil. Sci. Total Environ..

[146] Cavero J., Zaragoza C., Suso M.L., Pardo A. (1999). Competition between maize and Datura stramonium in an irrigated field under semi-arid conditions. Weed Res..

[147] Lee J.-S. (2011). Combined effect of elevated CO2 and temperature on the growth and phenology of two annual C3 and C4 weedy species. Agric. Ecosyst. Environ..

[148] Hanzlik K., Gerowitt B. (2012). Occurrence and distribution of important weed species in German winter oilseed rape fields. J. Plant Dis. Prot..

[149] Rodenburg J., Meinke H., Johnson D.E. (2011). Challenges for weed management in African rice systems in a changing climate. J. Agric. Sci..

[150] Noroozi S., Alizadeh H., Mashhadi H.R. (2016). Temperature influences postdispersal predation of weed seeds. Weed Biol. Manag..

[151] Rodenburg J., Riches C.R., Kayeke J.M. (2010). Addressing current and future problems of parasitic weeds in rice. Crop. Prot..

[152] Bailey S.W. (2004). Climate change and decreasing herbicide persistence. Pest Manag. Sci..

[153] Patterson D.T. (1995). Weeds in a Changing Climate. Weed Sci..

[154] Froud-Williams R. (1996). Weeds and climate change: Implications for their ecology and control. Asp. Appl. Biol..

[155] Holmsgaard P.N., Dealtry S., Dunon V., Heuer H., Hansen L.H., Springael D., Smalla K., Riber L., Sørensen S.J. (2017). Response of the bacterial community in an on-farm biopurification system, to which diverse pesticides are introduced over an agricultural season. Environ. Pollut..

[156] Liu L., Tang J., Zhong G., Zhen X., Pan X., Tian C. (2018). Spatial distribution and seasonal variation of four current-use pesticides (CUPs) in air and surface water of the Bohai Sea, China. Sci. Total Environ..

[157] Wang Q., Li C., Chen C., Chen J., Zheng R., Que X. (2018). Effectiveness of narrow grass hedges in reducing atrazine runoff under different slope gradient conditions. Environ. Sci. Pollut. Res..

[158] Székács A., Mörtl M., Darvas B. (2015). Monitoring Pesticide Residues in Surface and Ground Water in Hungary: Surveys in 1990–2015. J. Chem..

[159] Singh M., Pant G., Hossain K., Bhatia A.K. (2017). Green remediation. Tool for safe and sustainable environment: A review. Appl. Water Sci..

[160] Woodruff L.G., Cannon W.F., Eberl D.D., Smith D.B., Kilburn J.E., Horton J.D., Garrett R.G., Klassen R.A. (2009). Continental-scale patterns in soil geochemistry and mineralogy: Results from two transects across the United States and Canada. Appl. Geochem..

[161] Tirado M., Clarke R., Jaykus L., McQuatters-Gollop A., Frank J. (2010). Climate change and food safety: A review. Food Res. Int..

[162] Hailu F. (2016). Farmers perception of pesticide use and genetic erosion of landraces of tetraploid wheat (Triticum spp.) in Ethiopia. Genet. Resour. Crop. Evol..

[163] Miraglia M., Marvin H., Kleter G., Battilani P., Brera C., Coni E., Cubadda F., Croci L., De Santis B., Dekkers S. (2009). Climate change and food safety: An emerging issue with special focus on Europe. Food Chem. Toxicol..

[164] Elgueta S., Moyano S., Sepúlveda P., Quiroz C., Correa A. (2017). Pesticide residues in leafy vegetables and human health risk assessment in North Central agricultural areas of Chile. Food Addit. Contam. Part B.

[165] Mingo V., Lötters S., Wagner N. (2017). The impact of land use intensity and associated pesticide applications on fitness and enzymatic activity in reptiles—A field study. Sci. Total Environ..

[166] Škrbic B., Cvejanov J., Durišić-Mladenović N. (2007). Organochlorine pesticides and polychlorinated biphenyls in surface soils of Novi Sad and bank sediment of the Danube River. J. Environ. Sci. Health Part B.

[167] Kawahara J., Horikoshi R., Yamaguchi T., Kumagai K., Yanagisawa Y. (2005). Air pollution and young children’s inhalation exposure to organophosphorus pesticide in an agricultural community in Japan. Environ. Int..

[168] Tuncel S.G., Oztas N.B., Erduran M.S. (2008). Air and groundwater pollution in an agricultural region of the Turkish Mediterranean coast. J. Air Waste Manag. Assoc..

[169] Baker L.W., Fitzell D.L., Seiber J.N., Parker T.R., Shibamoto T., Poore M.W., Longley K.E., Tomlin R.P., Propper R., Duncan D.W. (1996). Ambient Air Concentrations of Pesticides in California. Environ. Sci. Technol..

[170] Gouin T., Shoeib M., Harner T. (2008). Atmospheric concentrations of current-use pesticides across south-central Ontario using monthly-resolved passive air samplers. Atmos. Environ..

[171] Fang Y., Nie Z., Die Q., Tian Y., Liu F., He J., Huang Q. (2017). Organochlorine pesticides in soil, air, and vegetation at and around a contaminated site in southwestern China: Concentration, transmission, and risk evaluation. Chemosphere.

[172] Ansara-Ross T., Wepener V., Brink P.V.D., Ross M. (2012). Pesticides in South African fresh waters. Afr. J. Aquat. Sci..

[173] Wang D., Singhasemanon N., Goh K.S. (2017). A review of diazinon use, contamination in surface waters, and regulatory actions in California across water years 1992–2014. Environ. Monit. Assess..

[174] Ben Salem F., Ben Said O., Aissa P., Mahmoudi E., Monperrus M., Grunberger O., Duran R. (2016). Pesticides in Ichkeul Lake–Bizerta Lagoon Watershed in Tunisia: Use, occurrence, and effects on bacteria and free-living marine nematodes. Environ. Sci. Pollut. Res..

[175] Adams R.M., McAdams B.C., Arnold W.A., Chin Y.-P. (2016). Transformation of chlorpyrifos and chlorpyrifos-methyl in prairie pothole pore waters. Environ. Sci. Process. Impacts.

[176] Mazlan N., Ahmed M., Muharam F.M., Alam M.A. (2017). Status of persistent organic pesticide residues in water and food and their effects on environment and farmers: A comprehensive review in Nigeria. Semin.-Cienc. Agrar..

[177] Kurwadkar S. (2017). Groundwater Pollution and Vulnerability Assessment. Water Environ. Res..

[178] Lai W.Y. (2017). Pesticide use and health outcomes: Evidence from agricultural water pollution in China. J. Environ. Econ. Manag..

[179] Guo H., Wang Y. (2004). Specific vulnerability assessment using the MLPI model in Datong city, Shanxi province, China. Environ. Earth Sci..

[180] Anderson B.S., Phillips B.M., Voorhees J.P., Deng X., Geraci J., Worcester K., Tjeerdema R.S. (2018). Changing patterns in water toxicity associated with current use pesticides in three California agriculture regions. Integr. Environ. Assess. Manag..

[181] Brauns B., Jakobsen R., Song X., Bjerg P.L. (2018). Pesticide use in the wheat-maize double cropping systems of the North China Plain: Assessment, field study, and implications. Sci. Total Environ..

[182] Clemow Y.H., Manning G.E., Breton R.L., Winchell M.F., Padilla L., Rodney S.I., Hanzas J.P., Estes T.L., Budreski K., Toth B.N. (2018). A refined ecological risk assessment for California red-legged frog, Delta smelt, and California tiger salamander exposed to malathion. Integr. Environ. Assess. Manag..

[183] Otalvaro J.O., Brigante M. (2017). Interaction of pesticides with natural and synthetic solids. Evaluation in dynamic and equilibrium conditions. Environ. Sci. Pollut. Res..

[184] Aravinna P., Priyantha N., Pitawala A., Yatigammana S.K. (2018). Use pattern of pesticides and their predicted mobility into shallow groundwater and surface water bodies of paddy lands in Mahaweli river basin in Sri Lanka (vol 52, pg 37, 2016). J. Environ. Sci. Health Part B-Pestic. Food Contam. Agric. Wastes.

[185] Rose C.E., Coupe R.H., Capel P.D., Webb R.M. (2018). Holistic assessment of occurrence and fate of metolachlor within environmental compartments of agricultural watersheds. Sci. Total Environ..

[186] Kole R., Banerjee H., Bhattacharyya A. (2001). Monitoring of market fish samples for Endosulfan and Hexachlorocyclohexane residues in and around Calcutta. Bull. Environ. Contam. Toxicol..

[187] Wang Y., Zhang S., Cui W., Meng X., Tang X. (2018). Polycyclic aromatic hydrocarbons and organochlorine pesticides in surface water from the Yongding River basin, China: Seasonal distribution, source apportionment, and potential risk assessment. Sci. Total Environ..

[188] Osten J.R.-V., Dzul-Caamal R. (2017). Glyphosate Residues in Groundwater, Drinking Water and Urine of Subsistence Farmers from Intensive Agriculture Localities: A Survey in Hopelchén, Campeche, Mexico. Int. J. Environ. Res. Public Health.

[189] Donald D.B., Syrgiannis J., Hunter F., Weiss G. (1999). Agricultural pesticides threaten the ecological integrity of northern prairie wetlands. Sci. Total Environ..

[190] Shakeri A., Mehrabi B. (2015). Potentially toxic elements and persistent organic pollutants in water and fish at Shahid Rajaei Dam, north of Iran. Int. J. Environ. Sci. Technol..

[191] Barron M., Ashurova Z.J., Kukaniev M.A., Avloev H.K., Khaidarov K.K., Jamshedov J.N., Rahmatullova O.S., Atolikshoeva S.S., Mamadshova S.S., Manzenyuk O. (2017). Residues of organochlorine pesticides in surface soil and raw foods from rural areas of the Republic of Tajikistan. Environ. Pollut..

[192] Zhang F., He J., Yao Y., Hou D., Jiang C., Zhang X., Di C., Otgonbayar K. (2013). Spatial and seasonal variations of pesticide contamination in agricultural soils and crops sample from an intensive horticulture area of Hohhot, North-West China. Environ. Monit. Assess..

[193] Wu L., Chang H., Ma X. (2017). A modified method for pesticide transport and fate in subsurface environment of a winter wheat field of Yangling, China. Sci. Total Environ..

[194] Andreu V., Picó Y. (2004). Determination of pesticides and their degradation products in soil: Critical review and comparison of methods. TrAC Trends Anal. Chem..

[195] Langenbach T., Mano D., Campos M.M., Cunha A.L., De Campos T.M. (2017). Pesticide dispersion by spraying under tropical conditions. J. Environ. Sci. Health Part B.

[196] Lee S., McLaughlin R., Harnly M., Gunier R.B., Kreutzer R. (2002). Community exposures to airborne agricultural pesticides in California: Ranking of inhalation risks. Environ. Health Perspect..

[197] de Jong F.M., de Snoo G.R., van de Zande J.C. (2008). Estimated nationwide effects of pesticide spray drift on terrestrial habitats in the Netherlands. J. Environ. Manag..

[198] Yera A.M.B., Nascimento M.M., da Rocha G.O., de andrade J.B., Vasconcellos P.C. (2020). Occurrence of Pesticides Associated to Atmospheric Aerosols: Hazard and Cancer Risk Assessments. J. Braz. Chem. Soc..

[199] Benka-Coker W., Hoskovec L., Severson R., Balmes J., Wilson A., Magzamen S. (2020). The joint effect of ambient air pollution and agricultural pesticide exposures on lung function among children with asthma. Environ. Res..

[200] Doan N.H., Duong H.T., Trinh H.T., Tanaka Y., Kadokami K. (2021). Comprehensive study of insecticides in atmospheric particulate matter in Hanoi, Vietnam: Occurrences and human risk assessment. Chemosphere.

[201] Pan D., He M., Kong F. (2020). Risk attitude, risk perception, and farmers’ pesticide application behavior in China: A moderation and mediation model. J. Clean. Prod..

[202] Sultana J., Syed J.H., Mahmood A., Ali U., Rehman M.Y.A., Malik R.N., Li J., Zhang G. (2014). Investigation of organochlorine pesticides from the Indus Basin, Pakistan: Sources, air–soil exchange fluxes and risk assessment. Sci. Total Environ..

[203] Colquhoun J., Heider D.J., Rittmeyer R.A. (2017). Seed Potato Growth and Yield as Affected by Mother Plant Exposure to Herbicides. Weed Technol..

[204] Hoai P.M., Sebesvari Z., Minh T.B., Viet P.H., Renaud F.G. (2011). Pesticide pollution in agricultural areas of Northern Vietnam: Case study in Hoang Liet and Minh Dai communes. Environ. Pollut..

[205] Fosu P.O., Donkor A.K., Ziwu C., Dubey B., Kingsford-Adaboh R., Asante I., Nyarko S., Tawiah R., Nazzah N. (2017). Surveillance of pesticide residues in fruits and vegetables from Accra Metropolis markets, Ghana, 2010–2012: A case study in Sub-Saharan Africa. Environ. Sci. Pollut. Res..

[206] Wanwimolruk S., Phopin K., Boonpangrak S., Prachayasittikul V. (2016). Food safety in Thailand 4: Comparison of pesticide residues found in three commonly consumed vegetables purchased from local markets and supermarkets in Thailand. PeerJ.

